# Dengue fever in a kidney transplant recipient with complicated clinical course: a case report

**DOI:** 10.1186/s13256-018-1790-0

**Published:** 2018-09-01

**Authors:** Ranga Migara Weerakkody, Dhammika Randula Palangasinghe, Eranga Sanjeewa Wijewickrama

**Affiliations:** 1 Department of Nephrology, Teaching Hospital, Jaffna, Sri Lanka; 2 University Medical Unit, Teaching Hospital, Karapitiya, Galle, Sri Lanka; 3 Department of Clinical Medicine, Faculty of Medicine, University of Colombo, 8 Kyinsey Road, Colombo, Sri Lanka

**Keywords:** Dengue fever, Renal transplant, Hepatitis B, Acute kidney injury, Pancytopenia

## Abstract

**Background:**

Dengue fever is the commonest mosquito-borne illness in the tropics and subtropics. Renal transplantation is one of the ever expanding modes of treatment of end-stage renal disease. Hepatitis B is a common infection in South and East Asia, but rare in Sri Lanka. Here we describe a recipient of a renal transplant with a stable graft, on antiviral treatment for hepatitis B infection, developing dengue superinfection and entering a complex clinical course. To the best of our knowledge this is the first report of such a case.

**Case presentation:**

A 59-year-old Sri Lankan woman developed acute renal failure and needed dialysis support; she had upper gastrointestinal bleeding that needed transfusions, pancytopenia, and a prolonged phase of thrombocytopenia. She eventually recovered from illness, and her renal functions returned to baseline levels. The differences in presentation, signs, symptoms, and mortality of renal transplant recipients infected with dengue fever from the general population are discussed, with possible reasons for altered presentation.

**Conclusions:**

Dengue superinfection in transplant recipients with hepatitis B infection can lead to management difficulties. The recovery can be slow as seen from this case, with prolonged thrombocytopenia.

## Background

Dengue fever (DF) is the commonest mosquito-borne illness in the tropics and subtropics. Many outbreaks have had devastating results over many years. In the majority of sufferers, DF is a subclinical infection, and in a minority, it presents with hemorrhagic manifestations. Solid organ transplant recipients have displayed a spectrum of clinical manifestations similar to their non-transplant counterparts. Immunosuppressive treatment plays a part in relatively less complications in transplant recipients than in their non-transplant counterparts, although fatal outcomes had been reported [1–4]. The course of the illness can be prolonged, in the form of: thrombocytopenia; unusual complications, such as graft dysfunction; or uncommon complications, like acute colitis [3, 5]. The occurrence of hepatorenal dysfunction in DF is being increasingly reported among previously healthy patients [6, 7] and, as a result, there is a novel enthusiasm on the pathogenesis of the disease.

Hepatitis B is an important, challenging infection among renal transplant recipients, although it is very rare among Sri Lankans due to safe blood products and immunization. The prevalence among patients with a renal transplant is estimated to approximately 0.1–0.4% [8]. There are no statistics about the Sri Lankan population, but in the authors’ experience, it is extremely rare. Here we report a case of DF in a post kidney transplant patient in a background of chronic active hepatitis B infection. Information on confirmed cases of DF among solid organ transplant recipients in the literature is limited, with very few fatalities; most of the fatalities occurring during the immediate post transplant period [2, 4]. Complete recovery of a post liver transplant recipient with DF was reported locally [9]. To the best of our knowledge this is the first reported case of DF in a post kidney transplant patient with chronic active hepatitis B infection.

## Case presentation

A 59-year-old Sri Lankan woman, recipient of an ABO matched, living donor kidney transplant performed in 1997, presented with a 3-day history of fever, a fall, mild headache, arthralgia, myalgia, abdominal pain, and progressive drowsiness. She was on tenofovir, azathioprine 75 mg daily, and prednisolone 5 mg daily. She denied respiratory, bowel, or urinary symptoms. We did not find a contact history of fever. She did not have any seizures during the illness. Her primary renal disease was membranous glomerulopathy diagnosed in 1989. She had diabetes and developed end-stage disease in 1996 and was commenced on hemodialysis. She received a transplant in 1997 and enjoyed an uncomplicated post-transplant period with creatinine values between 84 and 104 umol/L (50–110), and an excellent quality of life.

In 2013, she presented with progressive abdominal distension due to ascites and with stigmata of liver disease and was diagnosed as having cirrhosis. She was diagnosed as having hepatitis B infection, where the viral load was in the order of log 9, and renal function was within normal range. Serology for hepatitis C and human immunodeficiency virus (HIV) was negative. Therapy was initiated with lamivudine, but an inadequate response led to replacement with tenofovir 330 mg daily, which brought down the viral loads to order of log 2. She was very compliant in all her medications and did not have major adverse effects to any of her medications.

**Fig. 1:**
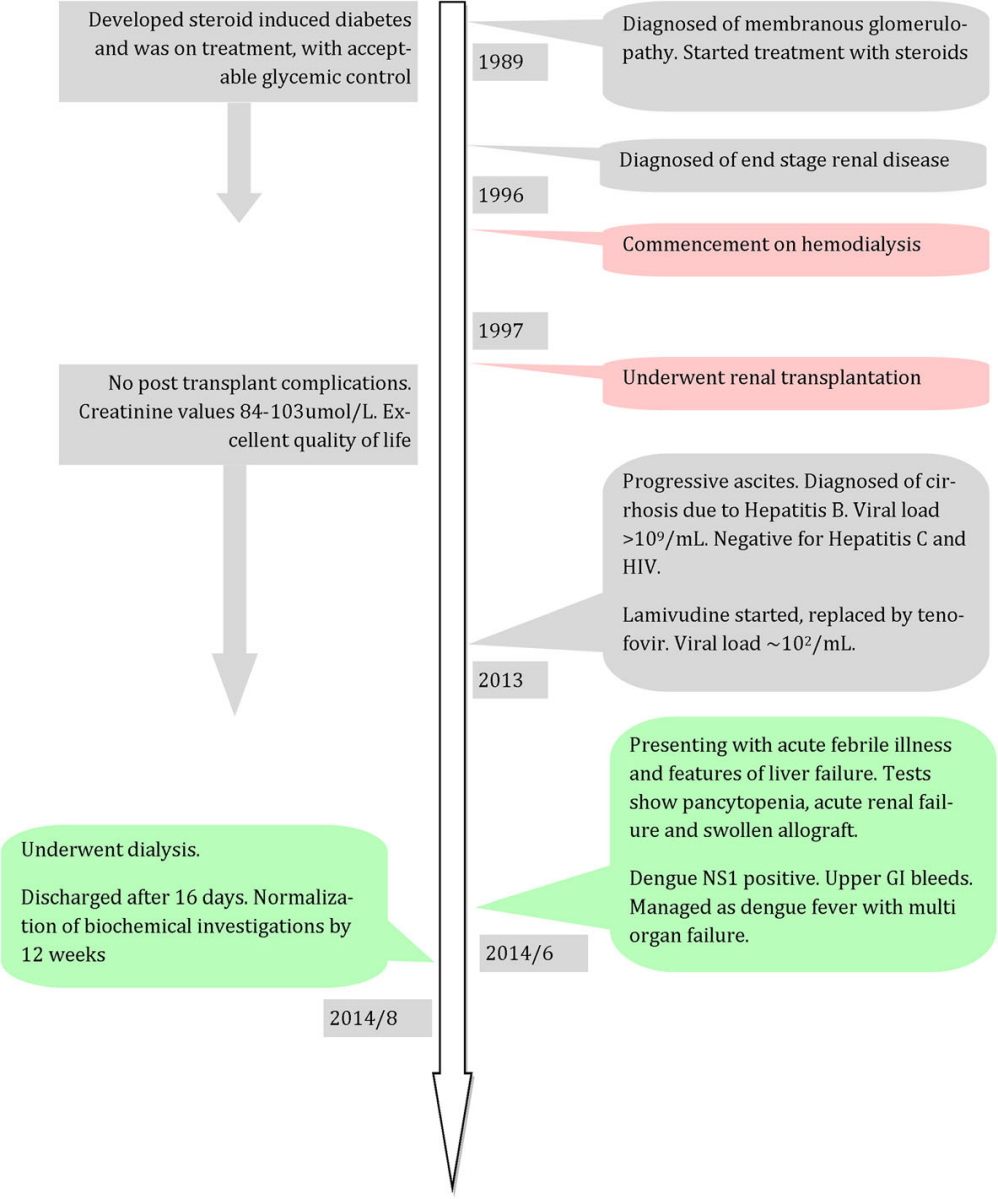
Case presentation in a timeline. *GI* gastrointestinal, *HIV* human immunodeficiency virus, *NS1* non-structural protein 1

**Fig. 2:**
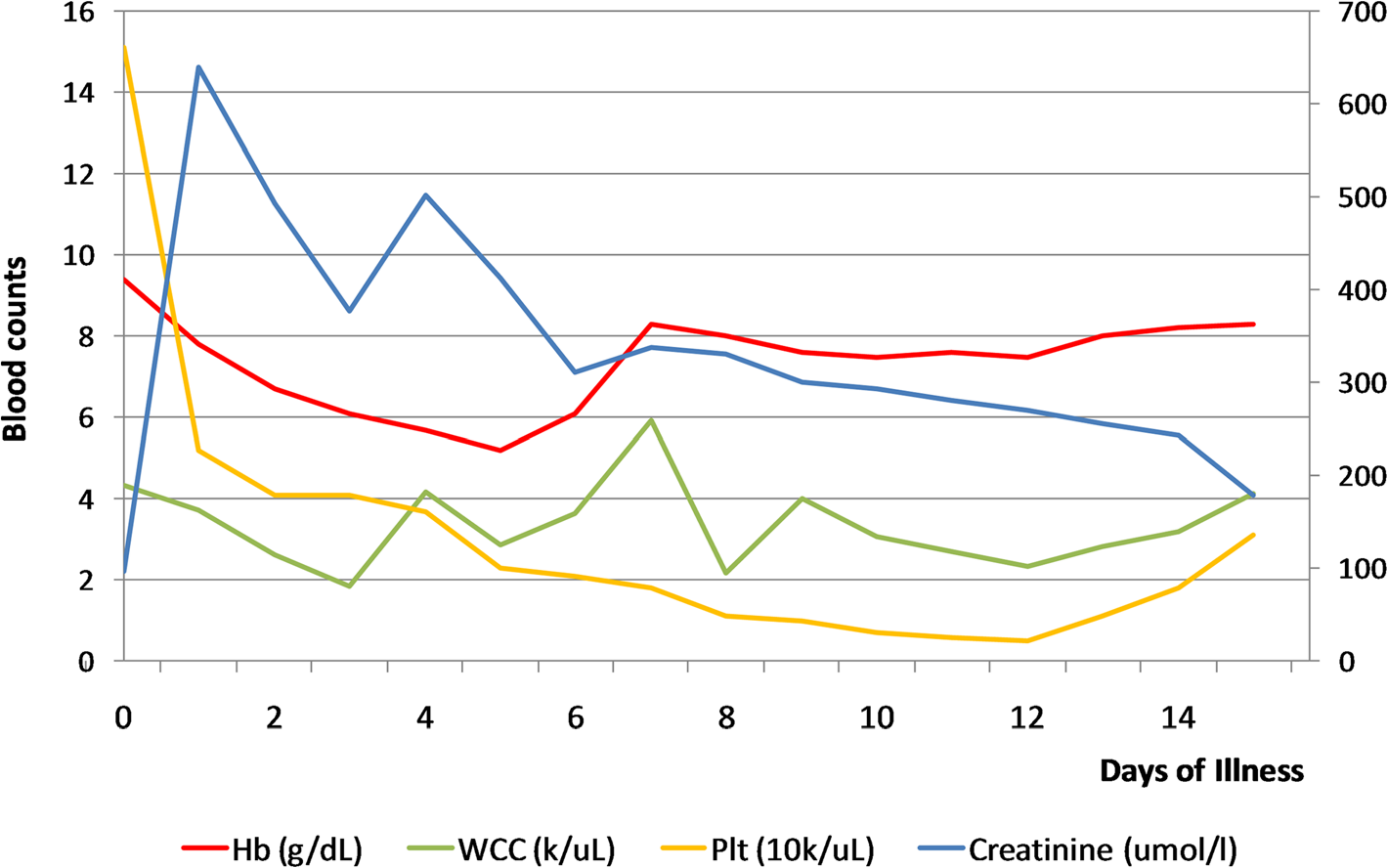
Changes in biochemical parameters over the course of hospital stay. *Hb* hemoglobin, *Plt* platelets, *WCC* white cell count

On examination she was drowsy, with Glasgow Coma Scale (GCS) of 12/15, and had flapping tremors. She was pale, anicteric, and was well hydrated. No skin rashes, cutaneous bleeding, or neck stiffness was noted. Her abdomen was soft on examination and tenderness noted in right iliac fossa overlying the graft. Her respiratory system and cardiovascular system examinations were clinically normal. Optic fundi were normal except for background diabetic retinopathy. Capillary blood sugar on admission was 7.7 mmol/L. Her initial investigations revealed pancytopenia on day 3 of the illness with hemoglobin (Hb) of 78 g/L (120–160), white cell count of 3.7 × 10^9^/L (3.5–12), and platelet count of 52 × 10^9^ /L (150–400). Blood picture showed normochromic normocytic red cells with reduced count, mild to moderate rouleaux formation, normal white cell count with hypersegmented and toxic-looking neutrophils. Severe thrombocytopenia was confirmed on the blood picture. We did not find any evidence of microangiopathic hemolytic anemia. Her C-reactive protein (CRP) level on admission was 108 mg/dL (< 6). Urine analysis showed 4–6 pus cells and 2–4 red cells, but no proteinuria. Serum creatinine was raised to 630 umol/L, from a background of 96 umol/L, checked 2 weeks ago on her routine clinic visit. Her serum creatinine phosphokinase level was normal. Urine and blood cultures were negative on day 3 of the illness. Non-contrast computed tomography (CT) of her brain was normal and a hip X-ray did not show evidence of any fracture. An ultrasound scan of her abdomen showed swollen kidney, with preserved corticomedullary demarcation, raising suspicions of acute renal parenchymal disease. She had no splenomegaly ultrasonically and portal vein diameter was 1.7 cm. She did not have oligo-anuria at that time. She developed metabolic acidosis, and with rising potassium levels, we decided to start renal replacement. Dengue non-structural protein 1 (NS1) antigen (day 2) was positive, but other viral studies such as cytomegalovirus and Epstein–Barr virus were negative. Subsequent blood counts revealed progressive decline in platelet and white cell counts. She developed an upper gastrointestinal (GI) bleed on day 6 (Hb, 52 g/L) and was supported with blood. From day 3 onward, of the illness, we empirically treated her with intravenously administered ceftriaxone for a suspected bacterial infection owing to her high CRP. Azathioprine and tenofovir were temporarily withheld due to possible marrow suppression and orally administered steroids doses were increased from 7.5 mg to 30 mg daily. Dengue IgM and IgG on day 6 were positive. Blood and urine cultures were sterile. Free fluid in the hepatorenal pouch was demonstrated on day 6 of the illness where she entered the critical phase. The lowest platelet count recorded was 5 × 10^9^/L. Her conscious level gradually improved and she was fully alert by day 5. She was never oligo-anuric during the period of illness. Upper GI endoscopy revealed portal gastropathy, antral gastritis, and non-bleeding small esophageal varices. We reintroduced tenofovir from day 5 onward. We had already taken her off azathioprine, but pancytopenia persisted. Intravenously administered ceftriaxone 1 g twice daily was continued up to 14 days, until CRP returned to normal. Her fluid management was monitored clinically, rather than according to the National Dengue guidelines, due to acute kidney injury (AKI). She was discharged on day 16 with creatinine normalized, but her liver enzymes and thrombocytopenia took 6 weeks to correct themselves. A review of this patient 12 weeks later revealed normal serum creatinine level and return of her blood counts to baseline. Figure 1 shows the case presentation in a timeline, while Fig. 2 describes the changes in the biochemical parameters. Table 1 describes pre-morbid, the most abnormal and convalescent values of important biochemical tests.

**Table 1:** Summary of laboratory investigations

Test	Units	Reference	Pre-illness	Most abnormal	Day#	Discharge	Follow up
Potassium	umol/L	3.5–5.1	4.1	6.3	2	3.3	4.0
CRP	umol/L	< 6	< 6	> 192*	2	6	< 6
ESR	mm/hour	< 20	24	128	2	60	12
Albumin	mg/dL	35–50	4.0	2.8	7	3.9	4.7
ALT	IU	< 40	27	116	10	67	28
AST	IU	< 40	40	267	10	160	39

*ALT* alanine aminotransferase, *AST* aspartate aminotransferase, *CRP* C-reactive protein, *ESR* erythrocyte sedimentation rate, *Day#* number of days after admission to the hospital, *Pre-illness* the most recent value before contracting the illness, *Follow up* is after 84 days, *CRP was measured using dilution method, and levels more than 32 times dilution expressed as > 192

## Discussion

DF occurs in outbreaks and has become the most widespread and important mosquito-borne illness in Sri Lanka, as well as in South and East Asia. It is mostly an asymptomatic or self-limiting infection; it has the ability to cause high morbidity and mortality. Although uncommon, complications like pericardial effusion, AKI, and acute colitis were reported with DF [1, 5, 10]. Despite high prevalence, DF is a rarely reported disease among patients who have had solid organ transplants. Similar to the non-transplant population, DF follows a benign course in most recipients of renal transplants. Severe dengue infection is rare [1, 10], but commoner than in non-transplant population [11]. The effects may be related to the relative balance between immunosuppression reducing the risk for antibody-enhanced complications, and reduced clearance of the viremia. Diminished T cell response can lead to atypical presentations in a transplant recipient [3, 4, 6]. Similar to the non-transplant population, early recognition will help to prevent or minimize complications. A systematic review by Weerakkody *et al.* showed that physical signs and symptoms differ from the general population [11]. Studies had shown that approximately 8.9% mortality was associated with dengue infection among renal transplant recipients [11]. In some cases mortality is a direct effect of bleeding [2], while others were due to secondary causes (for example, sepsis, type 1 respiratory failure) [7, 8].

Our patient had a prolonged period of thrombocytopenia running up to 16 days. Prolonged thrombocytopenia is a recognized feature of DF in patients with a renal transplant [1, 10]. Our patient had normal renal functions for 19 years post transplant up to this illness. The common causes of graft failure would be rejection, pyelonephritis, allograft nephropathy, and hepatorenal syndrome in this patient. Her urine analysis and sterile culture are not supportive of pyelonephritis, but high CRP and swollen allograft during imaging makes it a possibility. DF is known to cause renal dysfunction, sometimes to the extent of needing renal replacement [1, 3, 4]. However, elevated CRP is a known association with severe DF [12, 13]. We did not pursue a renal biopsy in the face of severe bleeding risk and rapid improvement in renal functions. Pre-renal AKI was also unlikely because she was not dehydrated and she never became oligo-anuric. Rejection is unlikely, given the spontaneous recovery without anti-rejection medication. Hepatorenal syndrome is a very likely cause, but oligo-anuria is the norm rather than an exception. DF as the cause for her AKI remains the plausible explanation. This patient had severe AKI which needed dialysis, but had a complete recovery of renal function of the allograft.

Several factors could have contributed to the pancytopenia in this patient, including azathioprine, tenofovir, acute viral illness, and chronic liver cells disease; however, normal blood counts before the febrile illness make this diagnosis unlikely. Leukopenia and thrombocytopenia are well-recognized features of DF, including the population of transplant recipients [1]. A fall in Hb and packed cell volume instead of the usual hemoconcentration during the critical phase can be associated with gut bleeding. However, true aplastic anemia has been described with DF, and some of these cases have been attributed to hemophagocytic syndrome [14] and others to bone marrow suppression [15, 16]. Our patient did not undergo a bone marrow biopsy; hence, it is impossible to differentiate bone marrow pathology from a peripheral pathology. However, a very low reticulocyte index of 0.2% indicated that the former is more likely. Aplastic anemia reported with dengue had been treated with intravenously administered immunoglobulins, steroids, and cyclosporine [15, 16]. Our patient was already on immunosuppressives; hence, the aplastic crisis could have become self-limited with the medications she was already receiving.

This patient was a particularly difficult case to manage due to multiple comorbidities such as cirrhosis, hepatitis B infection, acute renal failure which needed dialysis, pancytopenia, and immunosuppression. Management guidelines are not available on patients with such a complex clinical picture due to the rarity of the combinations of the disease. Accurate clinical assessment and objective-guided fluid management made management of this patient a success.

## Conclusion

Management of dengue in patients with comorbidities could be difficult. Dengue can cause severe allograft dysfunction, but it is reversible at least in many cases.

## Abbreviations

AKI: Acute kidney injury
CRP: C-reactive protein
CT: Computed tomography
DF: Dengue fever
GCS: Glasgow Coma Scale
GI: Gastrointestinal
Hb: Hemoglobin
HIV: Human immunodeficiency virus
NS1: Non-structural protein 1

## References

[1] Azevedo LS, Carvalho DB, Matuck T, Alvarenga MF, Morgado L, Magalhaes I, *et al.* Dengue in renal transplant patients: a retrospective analysis. Transplantation. 2007;84(6):792–4. PubMed PMID: 17893614. Epub 2007/09/26. eng 10.1097/01.tp.0000280547.91617.25 17893614

[2] Maia SH, Brasil IR, Esmeraldo Rde M, Ponte CN, Costa RC, Lira RA. Severe dengue in the early postoperative period after kidney transplantation: two case reports from hospital Geral de Fortaleza. Rev Soc Bras Med Trop. 2015;48(6):783–5. 10.1590/0037-8682-0205-2015 26676509

[3] Nasim A, Anis S, Baqi S, Akhtar SF, Baig-Ansari N. Clinical presentation and outcome of dengue viral infection in live-related renal transplant recipients in Karachi. Pakistan Transpl Infect Dis. 2013;15(5):516–25. PubMed PMID: 23890225. Epub 2013/07/31. eng 10.1111/tid.12114 23890225

[4] Prasad N, Bhadauria D, Sharma RK, Gupta A, Kaul A, Srivastava A. Dengue virus infection in renal allograft recipients: a case series during 2010 outbreak. Transpl Infect Dis. 2012;14(2):163–8. PubMed PMID: 22212524. Epub 2012/01/04. eng 10.1111/j.1399-3062.2011.00699.x 22212524

[5] Park SB, Ryu SY, Jin KB, Hwang EA, Han SY, Kim HT, *et al.* Acute colitis associated with dengue fever in a renal transplant recipient. Transplant Proc. 2008;40(7):2431–2. PubMed PMID: 18790257. Epub 2008/09/16. eng 10.1016/j.transproceed.2008.07.037 18790257

[6] Jain PK, Sharma AK, Agarwal N, Siddiqi MZ, Pawal P, Gaba R. A prospective clinical study of incidence of hepatorenal and hematological complications in dengue fever and management of symptomatic bleed in bundelkhand region of northern India with fresh whole blood. J Infect Dis Immun. 2011;3(7):124–33.

[7] Lima EQ, Gorayeb FS, Zanon JR, Nogueira ML, Ramalho HJ, Burdmann EA. Dengue haemorrhagic fever-induced acute kidney injury without hypotension, haemolysis or rhabdomyolysis. Nephrol Dial Transplant. 2006;22(11):3322–6. 10.1093/ndt/gfm431 17623717

[8] Kalia H, Fabrizi F, Martin P. Hepatitis B virus and renal transplantation. Transplantation. 2011;25(3):102–9. 10.1016/j.trre.2011.02.001 21530218

[9] Weerakkody RM, Palangasinghe DR, Dalpatadu KPC, Rankothkumbura JP, Cassim MRN, Karunanayake P. Dengue fever in a liver-transplanted patient: a case report. J Med Case Rep. 2014;8:378. 10.1186/1752-1947-8-378 PMC4244141 25412699

[10] Renaud CJ, Manjit K, Pary S. Dengue has a benign presentation in renal transplant patients: a case series. Nephrology (Carlton). 2007;12(3):305–7. PubMed PMID: 17498128. Epub 2007/05/15. eng 10.1111/j.1440-1797.2007.00785.x 17498128

[11] Weerakkody RM, Patrick JA, Sheriff MH. Dengue fever in renal transplant patients: a systematic review of literature. BMC Nephrol. 2017;18(1):15. 10.1186/s12882-016-0428-y PMC5237298 28086881

[12] Bodinayake CK, Weerarathna TP, Liyanage PLGC, LS Basnayake, editors. Value of C-reactive protein in assessing adverse outcomes of dengue fever. Annual Scientific Sessions; 2004. http://www.ruh.ac.lk/research/academic_sessions/2004_mergepdf/80-81.PDF. Accessed 25 Feb 2018.

[13] Chen CC, Lee IK, Liu JW, Huang SY, Wang L. Utility of C-reactive protein levels for early prediction of dengue severity in adults. Biomed Res Int. 2015;2015:936062. Epub 2015 Jul 12 10.1155/2015/936062 PMC4515279 26247033

[14] Jain D, Singh T. Dengue virus related hemophagocytosis: a rare case report. Hematology. 2008;13(5):286–8. 10.1179/102453308X316095 18854091

[15] Albuquerque PL, Silva Júnior GB, Diógenes SS, Silva HF, *et al.* Travel Med Infect Dis. 2009;7(2):118–2. 10.1016/j.tmaid.2009.01.001 19237145

[16] Ramzan MPS, Sachdeva A. Post-dengue fever severe aplastic anemia: a rare association. Hematol Oncol Stem Cell Ther. 2012;5(2):122–4. 10.5144/1658-3876.2012.122 22828377

